# Mitigating Aspiration Risk With Awake Intubation: A Case of Intrathoracic Stomach

**DOI:** 10.7759/cureus.73709

**Published:** 2024-11-14

**Authors:** Germano Carreira, Mariana Pascoal, Celine Ferreira

**Affiliations:** 1 Anesthesiology, Unidade Local de Saúde de Coimbra, Coimbra, PRT

**Keywords:** airway management, aspiration risk, awake fiberoptic intubation, awake intubation, fiberoptic intubation, hiatal hernia repair, endotracheal intubation

## Abstract

Hiatal hernias are common in the elderly and in most cases asymptomatic and no intervention is needed. Hiatal hernias can range from asymptomatic to an intrathoracic stomach, a rare but potentially life-threatening condition, characterized by the migration of the stomach into the thoracic cavity. Its need for urgent intervention presents a major concern for the anesthesiologist because it is associated with a high risk of aspiration.

This case report discusses the clinical presentation and anesthetic management of an intrathoracic full stomach proposed for urgent surgery. By presenting this case, we hope to enhance the understanding of preoperative management in patients with intrathoracic full stomach and the role of awake fiberoptic guided intubation as a valuable technique in emergency surgical settings. This case reinforces the importance of existing healthcare professionals with practice in managing similar clinical scenarios effectively.

## Introduction

The migration of the stomach into the thoracic cavity can occur due to various factors, including diaphragmatic hernia, trauma, or congenital abnormalities [1]. A hiatal hernia describes an enlarged diaphragmatic esophageal hiatus, through which the gastroesophageal transition migrates. In its maximum variant, the entire stomach and other abdominal organs can be shifted thoracically [2]. This abnormal positioning can lead to compression of vital structures, resulting in respiratory distress, gastrointestinal complications, and even cardiorespiratory compromise [3]. Symptoms of hiatal hernia are related to gastroesophageal reflux disease, but also to intrathoracic compression symptoms due to the intrathoracic dislodgement of the organs into the mediastinum, with the most dramatic presentation being ischemia of the herniated organs due to strangulation [2]. Its early diagnosis and prompt surgical intervention are essential in preventing further complications and preserving the patient's life. Presently, there are no recommendations regarding which surgical technique should be used for hiatal hernia correction, and the decision relies on the institutional experience in upper gastrointestinal surgery [2]. Laparoscopic surgery is a safe and effective treatment option for intrathoracic stomach, with or without gastric volvulus. It is associated with low rates of complications and recurrence. Self-reported patient data shows significant improvement in overall QoL after surgery for intrathoracic stomach [4].

While anesthesia is generally safe, respiratory complications such as anesthesia-related aspiration can be fatal. Occurring as often as one in every 2-3000 operations requiring anesthesia, almost half of all patients who aspirate during surgery develop a related lung injury, such as pneumonitis or aspiration pneumonia [5]. The need for emergency surgery and the presence of hiatal hernia are known predisposing conditions for aspiration in the perioperative period [5]. The majority of the patients who aspirated had either gastrointestinal obstruction or acute intraabdominal processes. Anesthesia care was frequently judged to be substandard [6].

Awake tracheal intubation (ATI) refers to any technique involving the placement of an endotracheal tube (ETT) in a non-anesthetized, spontaneously breathing patient capable of obeying commands. It is considered the gold standard for managing the predicted difficult airway and the risk of gastric aspiration [7]. Awake intubation is recommended for patients who are at high risk for difficult mask ventilation, particularly those who may be at an elevated risk for aspiration during the airway management process. A second benefit to this technique is that it may minimize aspiration risk because airway protective reflexes are maintained until just before the passage of an ETT [8]. Therefore, this approach allows for a controlled and meticulous intubation process, minimizing the risk of gastric content aspiration associated with general anesthesia’s induction and maintaining patient safety throughout the airway manipulation, which is essential in an intrathoracic stomach presenting for emergency surgery [9].

## Case presentation

A 78-year-old female (weight 72 Kg; height 170 cm) was admitted with retrosternal pain, of moderate intensity, without relief or aggravating factors, associated with nausea, vomiting, and dysphagia for liquids and solids. No other symptomatology was found. The patient had a history of controlled arterial hypertension, first-degree atrioventricular (AV) block, and hypothyroidism. Her current medications included alprazolam 0,5 mg PO once daily, levothyroxine 0.125 mg PO once daily, and lorazepam 1 mg PO at bedtime and she was allergic to fosfomycin. The airway evaluation showed a Mallampati I without any signs of a potentially difficult airway. Standard preoperative fasting was ensured.

**Figure 1:**
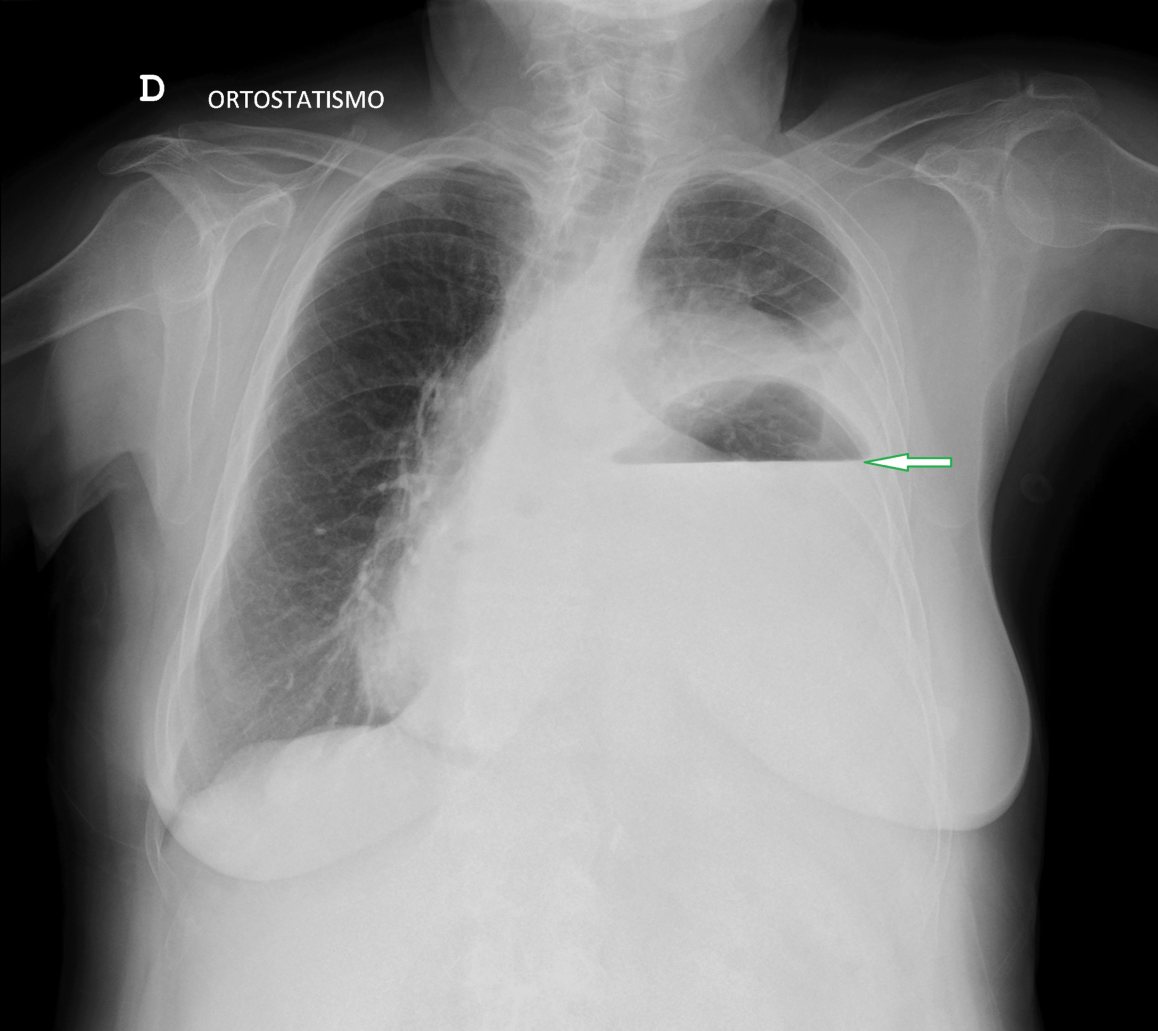
Chest X-ray revealing an intrathoracic gastric air-fluid level (green arrow)

**Figure 2:**
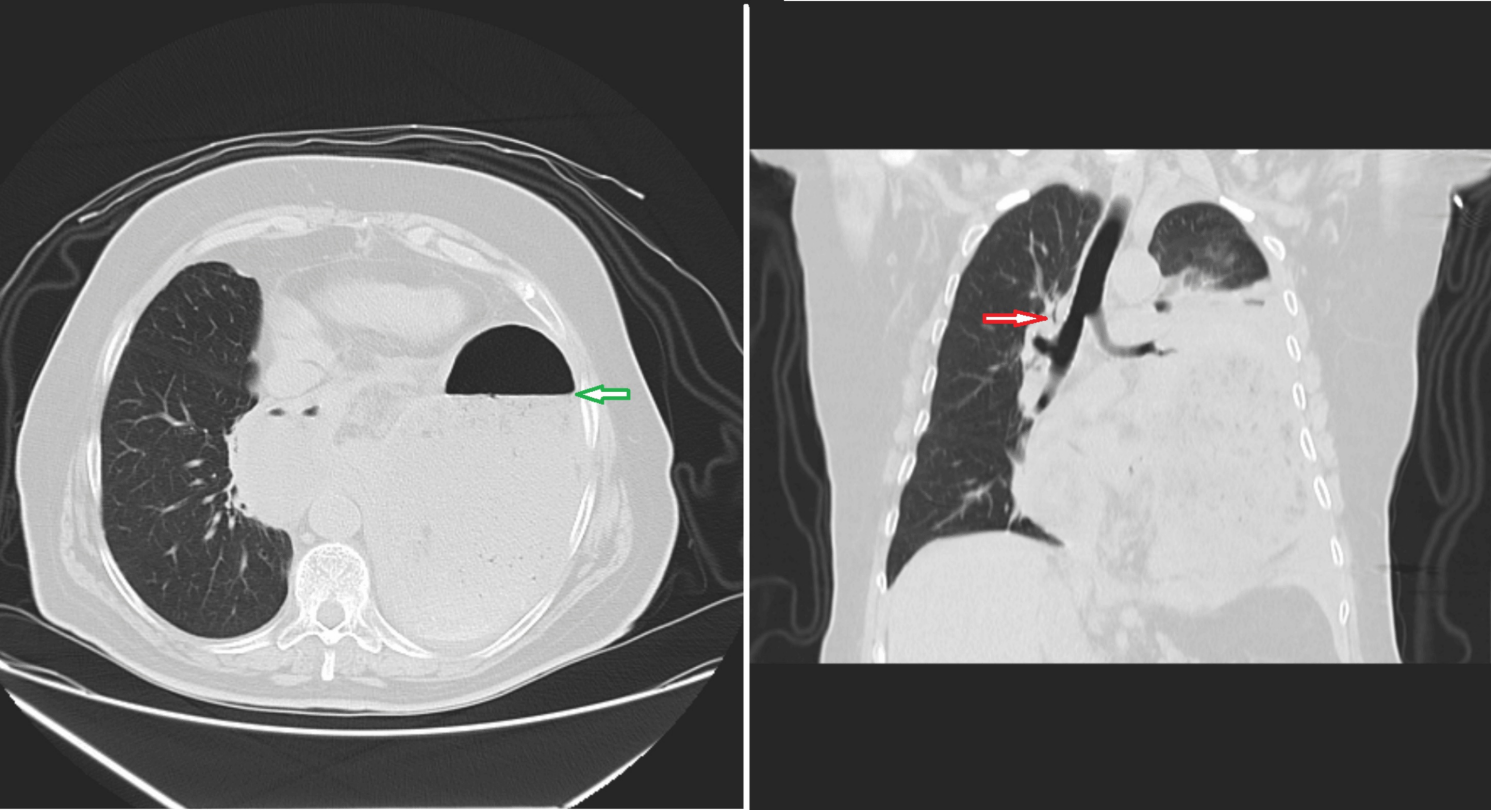
Thoracic CT scan showing an air-fluid level (green arrow) and right mediastinal shift (red arrow)

A 12-lead electrocardiogram showed a sinus rhythm, 75 bpm, compatible with first-degree AV block and occasional premature ventricular beat. High-sensitivity troponin levels turned out normal. The initial chest X-ray revealed an intrathoracic gastric air-fluid level (Figure 1), which was confirmed by a CT scan (Figure 2): a large air-fluid level within the left hemithorax, exerting pressure on the left lung and resulting in a mediastinal shift toward the right side, along with deviation of the trachea and heart.

In the emergency ward, multiple unsuccessful attempts to advance a nasogastric tube beyond a certain esophageal level were reported. Due to the urgency, a multidisciplinary team, comprising general surgeons, anesthesiologists, and gastroenterologists, was mobilized and the patient was proposed for endoscopic decompression under general anesthesia and subsequent surgical correction. Consent was obtained for the procedure.

The patient was premedicated with an intravenous proton pump inhibitor. Upon arrival to the operation room, the patient was fully conscious and cooperative and complained of mild chest discomfort. Standard ASA monitoring was used, and radial artery catheterization was performed for continuous invasive blood pressure. Heart rate (HR) was 75 beats/min, blood pressure (BP) was 110/62 mmHg, and pulse oximetry (SpO_2_) was 89% with a high concentration mask.

The mucosa of the oropharynx was topically anesthetized with 10% lidocaine and 100mcg fentanyl was administered intravenously. Minutes later, oral endotracheal intubation was performed using a fiberoptic bronchoscope while the patient was still breathing spontaneously and cooperatively. Upon visualization of the vocal cords, 5mL of 2% lidocaine was applied directly to the vocal cords and trachea. As the bronchoscope reached the trachea a seven-cuffed ETT was passed under direct visualization. The correct placement of the ETT was confirmed by observing bilateral chest rise, end-tidal CO_2_ detection, and visualization of the tube within the trachea. Subsequently, the induction was performed with 140 mg intravenous propofol, 2% sevoflurane was initiated for general anesthesia maintenance, and curarization was ensured with 30 mg of rocuronium. Catheterization of the right internal jugular vein was performed under ultrasound guidance without incidents.

The attempt at endoscopic decompression of the stomach and subsequent laparoscopic surgical reduction was unsuccessful. Therefore, the decision was to proceed to a midline laparotomy with a reduction of the entire hiatal hernia and retraction of the stomach into the abdominal cavity, which later revealed signs of distress. A chest drain was placed on the left side. Occasional premature ventricular beats, followed by ventricular tachycardia, were observed during hernia repair and ceased after the interruption of the maneuver.

The surgery lasted for one hour and blood loss was minimal. Anesthetic emergence was uneventful. The patient was transferred to the postanesthetic care unit and later discharged with no events.

**Figure 3:**
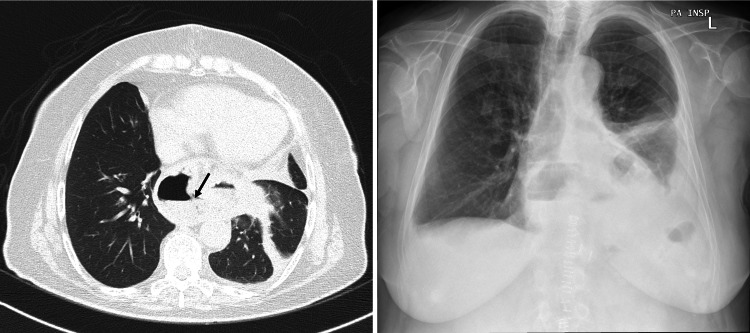
Re-evaluation with thoracic CT scan and chest X-ray showing a small sliding esophageal hiatal hernia (black arrow)

After being followed for up to three months, the patient was free of thoracic or abdominal discomfort, reflux, vomiting, or symptoms of heart failure. Meanwhile, the re-evaluation thoracic CT scan showed signs of previous surgical repair of a left diaphragmatic hernia, with moderate elevation of the left diaphragmatic hemi-dome and sliding esophageal hiatal hernia, with part of the stomach intra-thoracic (Figure 3).

## Discussion

The presented clinical case highlights the management of an intrathoracic full stomach with ATI to prevent potential respiratory complications. Intrathoracic stomach, resulting from a hiatal hernia, is a rare but significant condition. The abnormal positioning of the stomach within the thoracic cavity could possibly cause complications such as lung compression, resulting in respiratory distress. Associated symptoms such as dyspnea and chest pain or arrhythmia, including sinus tachycardia, atrial flutter, atrial fibrillation, extrasystole, and even cardiac arrest, are possible due to compression of the heart [3,10]. Immediate surgical intervention is often warranted to alleviate these symptoms and prevent further complications. The decision to proceed with urgent surgical repair was based on the patient's condition and the need for prompt intervention.

The choice to proceed with an urgent general anesthesia may bring some complications. General anesthesia frequently requires positive pressure ventilation. It causes a rise in intrathoracic pressure, which may compromise the already precarious hemodynamic status in large hiatal hernias due to posterior cardiac compression. The implications of hiatal hernia-induced cardiac distortion on arrhythmia pathogenesis are poorly recognized [11]. Spontaneous breathing maintains the diaphragm contractile force and prevents further tissue herniation and excessive chest pressure. It is estimated that over 50% of airway-related deaths in anesthesia are caused by peri-operative aspiration [12]. Aspiration pneumonia is caused by the pulmonary aspiration of gastric contents with an acidic pH, leading to the destruction of alveolar surfactant and causing progressive hypoxemia. This is a potentially fatal complication whose incidence increases in urgent surgery compared to elective surgery. Five prospective observational studies representing 793 emergency surgery patients showed that the incidence of "full stomach" was between 18% and 56% at the time of induction. A risk factor considered for a full stomach before emergency surgery was abdominal surgery [12]. The presence of gastrointestinal obstruction or hiatal hernia, along with the need for emergency surgery, were considered predisposing factors for aspiration during general anesthesia [5].

The decision to proceed with the urgent correction of a hiatal hernia may present significant aspiration risk. A large intrathoracic stomach may exhibit delayed gastric emptying, and it is a hazard for anesthesia induction. In fact, recent findings suggest that clinical practice modifications to preoperative assessment and anesthetic management of patients at risk for pulmonary aspiration may lead to improvement of their peri-operative outcomes [6].

The aspiration risk could call for the use of rapid sequence intubation (RSI) in a difficult airway, but it poses significant risks and challenges. RSI is a technique employed to secure a patient's airway swiftly, typically in emergency situations or when immediate intervention is required. The limited time available during RSI may lead to potentially suboptimal attempts at intubation, increasing the likelihood of complications such as esophageal intubation, dental or airway trauma, or aspiration of gastric contents [13]. ATI is a valuable option since the risk of gastric aspiration can be minimized. By keeping the patient conscious during intubation, the risks associated with the induction of general anesthesia, such as loss of protective airway reflexes and ventilatory compromise, are minimized, allowing continuous patient monitoring and immediate detection of any signs of respiratory distress or airway compromise, ensuring timely intervention if needed [9]. ATI allows the ability to secure the airway of a patient who maintains their intrinsic airway tone underpins the superior safety profile of ATI over techniques with the patient sedated heavily or anesthetized [14]. Both videolaryngoscopy and fiberoptic bronchoscopy can be used for ATI. In fact, videolaryngoscopes are being used more commonly. A recent systematic review showed that the intubation time was shorter when videolaryngoscopy was used instead of fiberoptic bronchoscopy, and it also seems to have a success rate and safety profile comparable to fiberoptic bronchoscopy [15]. The success of awake intubation relies on effective communication and collaboration between the anesthesia team and the patient. Patient counseling and perception of the procedure help to alleviate anxiety and build trust. Local anesthesia is administered to minimize discomfort during intubation, and careful titration of sedatives and analgesics ensures patient comfort.

The choice for ATI, in this case, was consistent with the understanding that it remains the “gold standard” technique in securing a definitive airway in conscious, self-ventilating patients with predicted or known difficult airways, and the procedure is associated with a low failure rate [16]. In this case, ATI was performed successfully, allowing the surgical team to proceed with the surgical plan previously mentioned and repair the intrathoracic stomach. By maintaining the patient's respiratory function and preserving airway reflexes, awake intubation played a crucial role in preventing potential respiratory complications during the procedure. It is important to acknowledge the limitations and potential risks associated with intubation under conscious sedation. This procedure requires expertise and leads to challenges such as patient anxiety, discomfort, or psychological distress. Continuous monitoring and readiness for unexpected complications are paramount in ensuring patient safety. Indeed, the practice of ATI remains low, at around 1% to 2% of all intubations. ATI, therefore, represents a skill that is key to patient safety but may not be practiced regularly by many anesthetists [16].

## Conclusions

This case of an intrathoracic full stomach, proposed for urgent surgical repair, highlights the significance of conscious intubation as a valuable technique in preventing respiratory complications and allows for timely intervention while maintaining the patient's cooperation. However, careful consideration of patient factors, collaboration between the surgical and anesthesia teams, and individualized decision-making are essential in determining the most appropriate approach for each patient.

Careful perioperative management and optimized multidisciplinary teams are the success factors in the management of rare conditions. In addition, sufficient planning and preserving spontaneous breathing during endotracheal intubation plays an essential role. Further studies and experiences will contribute to refining the management strategies for cases of intrathoracic stomach and optimizing patient outcomes.
